# The strategic uses of collagen in adherent cell cultures

**DOI:** 10.1002/cbin.11966

**Published:** 2022-11-24

**Authors:** PinFen Chua, William K. Lim

**Affiliations:** ^1^ Department of Paraclinical Sciences, Faculty of Medicine and Health Sciences Universiti Malaysia Sarawak Kota Samarahan Sarawak Malaysia

**Keywords:** cell adhesion, culture technology, extracellular matrix, light microscopy, proteoglycans/structural proteins

## Abstract

The culture of adherent mammalian cells involves adhesion to the tissue culture vessel. This requires attachment factors from serum and/or a suitable substrate on the vessel surface. Some cells require collagen or other substrates to promote neurite outgrowth, differentiation or growth. However, laboratories often lack guidance on the selection and/or optimisation of collagen. We model such selection/optimisation work in the PC12 neuronal cell line. PC12 (NS‐1 variant) cells require a substrate for adherence. Comparing cell attachment against a series of substrates, we found collagen IV to be optimal. We show by comparison of morphology against a range of concentrations that 10 µg/ml is sufficient for supporting cell attachment, and also differentiation. PC12 cells from Riken Cell Bank do not require a substrate for routine culturing but only for differentiation. As all substrates supported attachment equally well, we used a novel serum‐free approach and identified collagen IV as its preferred substrate. For these cells, Dulbecco's modified eagle's medium but not Roswell Park Memorial Institute (RPMI) media supports normal cell attachment. However, coating with collagen IV enabled the cells to grow equally well in RPMI. Hence the strategic use of collagen is essential in laboratories working with anchorage‐dependent cell lines.

## 1 INTRODUCTION

Cell culture is one of the most important techniques in cellular and molecular biology. Its usage spans drug development, vaccine production, tissue regeneration and genetic engineering. Cell lines are often used in preliminary studies while primary cultures, which better represent in vivo conditions, are used in confirmatory studies (Phelan & May, 2017). The homogeneity of clonal cell lines in well‐defined culture systems allows consistent and reproducible data without the confounding effects inherent to in vivo systems (Segeritz & Vallier, 2017).

In most tissues of the body, cells are surrounded and anchored to a porous network of cell‐secreted fibrous proteins and proteoglycans called extracellular matrix (ECM) (Bandzerewicz & Gadomska‐Gajadhur, 2022). The ECM provides structural support to tissues and stores growth and other secreted factors that can bind cellular receptors to regulate cellular behaviour and function. Integrins are a major class of cell adhesion molecules which are transmembrane receptors connecting cells to other cells and the ECM. Binding of ECM to integrins can initiate intracellular signalling that alters cytoskeletal organisation and modulate cell morphology, spread, growth, migration and viability (Muncie & Weaver, 2018).

Although anchorage‐dependent cells can be cultured on a glass or plastic (most commonly polystyrene) surface, they mostly do not adhere directly, but to attachment factors adsorbed to these surfaces. These factors are either secreted by the cells or present in the serum added to the culture media. Nevertheless, cell lines such as primary cells adhere poorly to polystyrene vessels including those surface‐treated for cell culture. Some laboratories culture cells on microplates for assays that involve washing steps, and find the cells dissociate upon washing. There are also experiments needing serum‐free media, and thus lack certain attachment and spreading factors. Though PC12 cells from Riken Cell Bank (Riken) can be cultured on normal tissue culture vessels, a substrate coating is advised for differentiation (Riken, personal communication). For all these reasons and also to maintain cells in a more native state in vitro and mimic in vivo behaviour, the tissue culture vessels can be coated with ECM components or synthetic polymers which can influence cell adhesion, morphology, growth, lifespan, migration, differentiation and neurite outgrowth (Kleinman et al., 1987).

Collagens, the most widely used ECM component coating in cell culture, is commercially available in various forms, along with other ECM proteins such as fibronectin and laminin. They are from animal or human sources, or recombinant. Poly ‐l‐lysine and poly‐d‐lysine are poly amino acids applied to tissue culture vessels to increase positively charged sites for better cell adhesion. However, laboratories often lack guidance on the selection and/or optimisation of these substrates. We model such selection/optimisation work using the PC12 neuronal cell line. PC12 was established from a rat pheochromacytoma (Greene & Tischler, 1976) and served neurobiology researchers for over 40 years. When treated with nanomolar concentrations of nerve growth factor (NGF), these cells stop proliferating, extend neuritic processes and take on properties of mature sympathetic neurons (Fujii et al., 1982). Various studies had attempted to identify an optimal substrate for PC12 cells but with a limited number of substrates, using no or only one type of collagen (Akeson & Warren, 1986; Orlowska et al., 2017; Tomaselli et al., 1987; Wiatrak et al., 2020). We reported that different variants of PC12 cells have differing requirements for adhesive substrate (Chua & Lim, 2021). Among them, the NS‐1 variant (NS‐1) requires a substrate for adherence.

Most laboratories using common adherent mammalian cell lines as a research tool perform two‐dimensional (2D) cell culture for one or more cell lines. Where an adhesive substrate is required, the most rapid and economical approach is to apply a single coating of a suitable substrate. Using phase contrast microscopy of NS‐1 cells as the model, we show how substrates can be compared to identify the optimal one. The optimum concentration is then determined for cost‐effectiveness. Where all substrates appear to support cell morphology equally, we demonstrate a novel serum‐free approach to determine the optimal substrate. Last, we illustrate a strategic use of collagen to support an otherwise unsuitable tissue culture media for a cell line.

## 2 MATERIALS AND METHODS

### 2.1 Reagents and chemicals

Cell culture medium (Dulbecco's modified eagle's medium [DMEM] high glucose and Roswell Park Memorial Institute [RPMI] 1640), horse serum and bovine serum albumin (BSA) fraction V (7.5%) were from Gibco. Foetal bovine serum was from Hyclone. Collagen I (Catalogue number 5005, Lot number 7503) and collagen IV (Catalogue number 5022, Lot number 7543) were from Advanced Biomatrix. Collagen I is prepared from collagen extracted from bovine hide and collagen IV is isolated from human placenta and is purified using a multi‐step process according to the manufacturer's product information. Laminin (Catalogue number CC095, Lot number 3062748), poly‐d‐Lysine (Catalogue number A‐003‐E, Lot number 80802‐1) and poly‐l‐Lysine (Catalogue number A‐005‐C, Lot number 3061974) were from Merck. The purified mouse laminin is prepared from Engelbreath‐Holm‐Swarm sarcoma according to the manufacturer's product information. Insulin from bovine pancreas, apo‐transferrin from human, ethanolamine, β‐mercaptoethanol, sodium selenite and other chemicals were from Sigma Aldrich.

### 2.2 Cell culture

Neuroscreen‐1 (NS‐1) cells, a variant of PC12 cells (formerly from Cellomics), were a generous gift from Dr Yves Le Dréan, University of Rennes 1, France. PC12 (originator: L. Greene and A. Tischler) RCB0009 cells (Riken) were obtained from Riken Cell Bank. NS‐1 cells and PC12 cells (Riken) were cultured in DMEM supplemented with 15% horse serum and 2.5% foetal bovine serum. Cells were incubated in a 37°C humidified CO2 incubator. Cells, passaged between 3 and 13 passages, were used for the experiments.

### 2.3 Cell morphology in different substrata

NS‐1 cells and PC12 (Riken) cells were cultured as described in ‘Cell Culture’ and were seeded into six‐well plates, uncoated and coated with 90 µg/ml collagen I, 10 µg/ml collagen IV, 50 µg/ml poly‐d‐lysine, 50 µg/ml poly‐l‐lysine or 10 µg/ml laminin. Cells were incubated in a 37°C humidified CO_2_ incubator. Cell morphology was assessed after 24 h incubation using an Olympus IX71 inverted microscope, and images were captured with an Olympus DP72 camera and examined using Cell^v^F imaging software (Olympus). Cell images were analysed using ImageJ 1.51 k software (NIH). All the cells (at least 50) in the image were counted. Cells were examined for ‘spread’ morphology and the cell was counted as one with spread morphology if the whole cell body is phase‐dark (Humphries, 1998). The percentage of cells with spread morphology is shown in the equation (Stockton & Jacobson, 2001). All experiments were repeated three times.

$$\mathrm{percentage}\;\mathrm{of}\;\mathrm{cells}\;\mathrm{with}\;\mathrm{spread}\;\mathrm{morphology}(\%)=\frac{\mathrm{number}\;\mathrm{of}\;\mathrm{cells}\;\mathrm{with}\;\mathrm{spread}\;\mathrm{morphology}}{\mathrm{total}\;\mathrm{number}\;\mathrm{of}\;\mathrm{cells}}\;x\;100$$

### 2.4 Cell morphology in different collagen IV concentrations

NS‐1 cells were seeded in a six‐well plate coated with 3, 10 and 30 µg/ml collagen IV and incubated for 24 h. Cell morphology was observed using an Olympus IX71 inverted microscope and images were captured with an Olympus DP72 camera and examined using Cell^v^F imaging software (Olympus).

### 2.5 Serum‐free supplemented media preparation

Serum‐free media with supplement was prepared by modification of a published serum‐free media for PC12 cells (Ohnuma et al., 2006). Six supplements (30 µg/ml insulin, 10 µg/ml apo‐transferrin, 500 µg/ml BSA, 10 µM ethanolamine, 10 µM β‐mercaptoethanol and 10 nM sodium selenite) were added to the serum‐free DMEM media for PC12 cells (Riken) in wells coated with different substrata.

### 2.6 Adaptation of cells to serum‐free supplemented DMEM media and RPMI media with serum

Adaptation of PC12 (Riken) cells cultured in DMEM in full serum to serum‐free DMEM with supplements as described in ‘Serum‐free supplemented media preparation’ was carried out according to the media adaptation protocol in the ATCC Animal Cell Culture Guide (ATCC Animal Cell Culture Guide, 2014). In brief, the cells were cultured in two flasks. One was in DMEM media with serum as control and the other in a ratio of 25% serum‐free supplemented media and 75% media with serum. Cell confluence rate and morphology were monitored to ensure similarity. The proportion of serum‐free DMEM with supplements increased progressively (to 50% and 75%) with each cell passaging. The cells were cultured in 75% serum‐free supplemented DMEM media for three passages before the experiment. Adaptation from DMEM media with serum to RPMI media with serum was similar as described. Each adaptation was repeated three times.

### 2.7 PC12 (Riken) cell in RPMI media uncoated versus collagen IV coated

PC12 (Riken) cells in DMEM media were adapted to RPMI media (both in 15% horse serum and 2.5% foetal bovine serum) using uncoated T25 flask or 10 µg/ml collagen IV‐coated T25 flask. Adaptation of cell culture was as described in ‘Adaptation of cells from full serum media to serum‐free supplemented media and RPMI media’. After adaptation, cells in the uncoated and collagen IV‐coated flask were passaged three times before cell observation. Cell morphology was assessed after a 2 to 3‐day incubation using an Olympus IX71 inverted microscope and images were captured with an Olympus DP72 camera and examined using Cell^v^F imaging software (Olympus).

### 2.8 Statistical analyses

All data were analysed using GraphPad Prism 9 software. Data sets were tested for statistical significance using one‐way analysis of variance (ANOVA) followed by post‐hoc Tukey's comparison test for comparing three or more data groups. A *p* value less than .05 was considered statistically significant. Data are reported as mean ± standard error of mean of at least three independent repeats.

## 3 RESULTS

### 3.1 Collagen IV is optimal for NS‐1 cells

**Figure 1:**
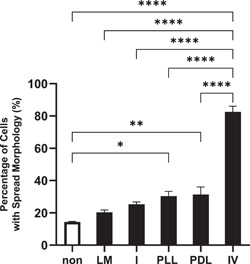
Percentage of cells with spread out morphology in NS‐1 cells in Dulbecco's modified eagle's medium (DMEM) with 15% horse serum and 2.5% foetal bovine serum. NS‐1 cells were seeded into six‐well plates with the wells ei‐ther uncoated (non) or coated with laminin (LM) collagen I (I), poly‐l‐lysine (PLL), poly‐d‐lysine (PDL), or collagen IV (IV). Cell morphology was observed after 24 h as described in Section 2. Percentages of the number of cells with spread‐out morphology over the total number of cells in the respective wells are shown. The data shown are the mean ± standard error of mean of three independent experiments. Statistical significance is shown as *p < .05, **p < .01, ****p < .0001 using one‐way analysis of variance.

NS‐1 cells, a PC12 variant with an adherent phenotype, require a substratum coating for cell adhesion, growth and differentiation. Optimisation of adhesive sub‐strate for PC12 cells reported so far have involved limited substrates, and none in the NS‐1 variant. We determined the optimal adhesive substrate of NS‐1 cells by analysing cells with spread morphology against five substrates (collagen I, collagen IV, laminin, poly‐d‐lysine and poly‐l‐lysine), as described in ‘Material and methods’. Figure 1 showed that 24 h after seeding, more than 80% ± 3.5% of NS‐1 cells in collagen IV‐coated plate had spread morphology. In contrast, only around 20.3% ± 1.5% to 31.3% ± 4.7% of cells had spread morphology in other substrates (one‐way ANOVA *p* < .0001). The experiment was repeated three times. Hence collagen IV is the optimal substrate for NS‐1 cell attachment.

### 3.2 Collagen IV at 10 µg/ml is sufficient for NS‐1 cells

**Figure 2:**
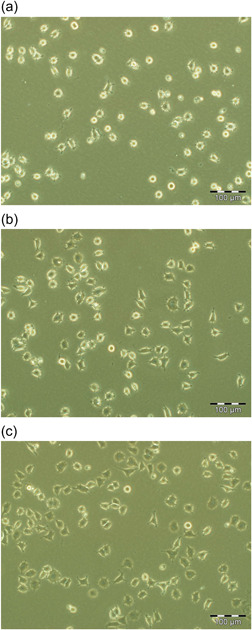
Cell morphology in different concentrations of collagen IV. NS‐1 cells were cultured in Dulbecco's modified eagle's medium (DMEM) media with 15% horse serum and 2.5% foetal bovine serum in a T25 flask as described in the Section 2. Cells were seeded into six‐well plate with each well coated with 3 µg/ml (a), 10 µg/ml (b) or 30 µg/ml (c) collagen IV respectively. After 24 h of incubation, representative phase contrast photomicrographs were taken. (a) Show a majority of rounded, loosely attached phase‐bright cells. (b) Shows a majority of spread out, firmly attached phase‐dark cells. (c) Shows almost all the cells are spread out and strongly attached. Scale bar (a, b, c) 100 µm.

To investigate the concentration of collagen IV on attachment of NS‐1 cells, we used 3, 10 and 30 µg/ml collagen IV‐coated plate for NS‐1 cell seeding. After 24 h incubation, the cell morphology is as shown in Figure 2. Most NS‐1 cells in the 3 µg/ml collagen IV‐coated well were round, phase‐bright and weakly attached, as shown in Figure 2a. Figure 2b (10 µg/ml) and Figure 2c (30 µg/ml) showed a majority of NS‐1 cells were well attached with spread out morphology and phase‐dark. Hence 10 µg/ml is sufficient for supporting cell attachment.

### 3.3 Collagen IV is the preferred substrate for PC12 cells (Riken)

**Figure 3:**
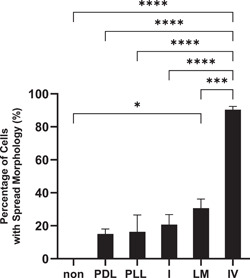
Percentage of cells with spread out morphology in PC 12 (Riken) cells in serum‐free supplemented media. PC12 (Riken) cells were adapted from full serum media as described in Section 2. Cells were seeded into six‐well plates with the wells either uncoated (non) or coated with laminin (LM) collagen I (I), poly‐l‐lysine (PLL), poly‐d‐lysine (PDL), or collagen IV (IV). Cell morphology was observed after 24 h as described in Section 2. Percentages of the number of cells with spread‐out morphology over the total number of cells in the respective wells are shown. The collagen IV‐coated well had the highest percentage of cells with spread‐out morphology. The data shown are the mean ± standard error of mean of three independent experiments. Statistical significance is shown as *p < .05, ***p < .001, ****p < .0001 using one‐way analysis of variance.

For culturing of PC12 cells (Riken), we found all substrates tested could support spread morphology in more than 81% of cells (data not shown). However, a substrate is required for differentiation. To determine the optimal substrate for use in differentiation, we used a novel approach by first adapting the cells to a serum‐free media added with chemically‐defined supplement. Subsequently, the different substrates were compared for supporting cell attachment. The percentage of cells with spread morphology using collagen IV was 90.3% ± 2.0% (one‐way ANOVA, *p* < .0001), as shown in Figure 3. Other substrates had markedly lower percentages of cells with spread morphology of 15.0% to 30%. The experiment was repeated three times. This approach enabled collagen‐IV to be identified as the preferred substrate for PC12 cells (Riken).

### 3.4 Collagen IV enabled change of media from DMEM to RPMI

**Figure 4:**
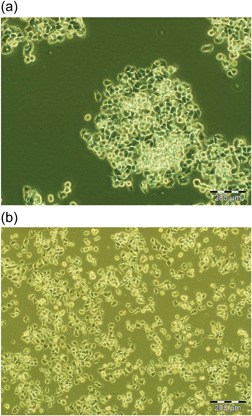
PC12 (Riken) cells in uncoated versus collagen IV‐coated flask. PC12 (Riken) cells were adapted from Dulbecco's modified eagle's medium (DMEM) to Roswell Park Memorial Institute (RPMI) (both in 15% horse serum and 2.5% foetal bovine serum) as described in the Section 2. Cells were seeded into an uncoated T25 flask and a 10 µg/mL collagen IV‐coated flask. Representative phase contrast images are shown after three passages in the RPMI media and imaged after 2–3 days of seeding. (a) PC12 (Riken) cells in uncoated T25 flask containing RPMI media are rounded, phase‐bright and clumped. (b) PC12 (Riken) cells in collagen IV‐coated T25 flask in the same RPMI media are spread out, phase dark and attached well. Scale bar 200 µM.

PC12 cells (Riken) cultured in DMEM could not attach well to an uncoated flask when adapted to RPMI media in full serum (Chua & Lim, 2021). To investigate the utility of collagen IV in supporting this change of media, we adapted the cells to RPMI and full serum media in a collagen‐IV coated flask. Figure 4b showed that the cells attached well to the collagen IV‐coated flask, with a ‘spread out’ morphology. In contrast, when a non‐coated flask was used, the cells were rounded, clumped and loosely attached (Figure 4a).

## 4 DISCUSSION

Two‐dimensional cell culture has been used since the 1900s and remains the most common cell culture platform. 3D cell culture is increasingly used, particularly in specific applications involving cancer cells, stem cells and drug discovery (Jensen & Teng, 2020). However, laboratories culturing only common adherent mammalian cell lines still only require 2D cell culture, and where necessary, an adhesive substrate for the tissue culture vessels. In this report we show how substratum coating can be optimised and used strategically. We identified the optimal substrate by comparison attachment of cells grown on different commonly available substrates. We tested poly‐l‐lysine and poly‐d‐lysine, both positively charged polymers that can change the negatively‐charged surfaces of tissue culture‐treated polystyrene vessels to positive for better attachment to negatively‐charged ions of cell membranes. At the same time, this coating can capture beneficial factors secreted by cells or present in serum (Stearns et al., 2016). We also tested collagen I, collagen IV and laminin, all components of the ECM. In contrast to ECM in vivo which is a 3D network surrounding the cells, there is only a single protein layer presented to the cells in vitro. Cell surface receptors such as the integrin family are able to bind specific ECM molecules. Cell‐matrix adhesions via integrins can trigger intracellular signalling that facilitates cell attachment, spread, survival and differentiation.

While all the substrates tested improved cell attachment over uncoated control, collagen IV exhibited a three to four‐fold bigger effect compared to the other substrates. This enhancement of cell attachment by collagen IV could be due to its direct binding to cells. While type I collagen is the most abundant collagen in the body, type IV occurs primarily in the basement membranes, which is the ECM layer adjacent to the cells (Bandzerewicz & Gadomska‐Gajadhur, 2022). Basement membranes are involved in cell adhesion, migration and are a reservoir of growth factors and enzymes (Khoshnoodi et al., 2008). Type IV collagen is known to interact with a large variety of cells, mostly via the integrin family of cell adhesion molecules. Integrin α1β1 and α2β1 have been reported to bind to collagen IV (Vandenberg et al., 1991). Another possible mechanism by which collagen IV promotes cell attachment is by binding to and forming a reservoir of cell‐secreted or serum factors that promote cell‐matrix adhesion and cell spread via binding to cell surface receptors.

We showed the identification of the one optimal substrate for use in a cell line. However, it is also possible to use two substrates. Orlowska et al showed a single coating of laminin or poly‐l‐lysine alone did not support attachment of PC12 cells as well as the combination of poly‐l‐lysine with laminin (Orlowska et al., 2017). However, using more than one substrate is costlier and involves a more complex coating procedure. Hence if a single substrate coat is sufficient for the cells, it will be more economical and rapid. We performed a dose–response experiment to determine the lowest effective concentration of the substrate. However, at high substrate concentration it is possible to observe patches of gel‐like regions under the microscope where cellular features are harder to distinguish (data not shown). Hence use of the lowest sufficient substrate concentration is both economical and practical.

We coat tissue culture vessels with substrate before use. When stored at 2–8°C, the coated vessels can retain their activity for at least 3 months. It is possible to purchase pre‐coated vessels, but these are costlier and have an expiry date. We compared commercially available collagen IV‐precoated vessels with those we coated at the usual concentration. When used in a disease model where cells die without intervention, the cells in the precoated vessels mostly survived (data not shown). This could be due to a difference in the collagen quality, concentration or unknown additives. Hence although precoated vessels can reduce the workload of coating, it is disadvantageous because of its higher cost, fixed expiry date and inability to modify the coating in accordance with the type of experiment.

Where the optimal substrate cannot be distinguished by comparing cell morphology in full serum media, we demonstrated a novel approach utilising serum‐free media added with a chemically‐defined supplement. This will preclude any serum factors that is supporting cell adherence without the need for substrate. Serum proteins involved in cell adhesion include laminin, fibronectin, vitronectin and thrompospondin (Yildirim, 1998). We also showed the use of an adhesive substrate to facilitate conversion to a different cell media. At times cells may not adhere in a new media despite using a media adaptation protocol. This can happen if the new media has a lower level of calcium or magnesium ions which are needed for cell attachment. In such cases, use of an adhesive substrate such as collagen can facilitate successful adaptation to a new media. The approaches and results from this work should provide useful models for laboratories in the strategic use of collagens in cell culture.

## AUTHOR CONTRIBUTIONS

William K. Lim conceptualised the study, acquired funding and supervised the project. PinFen Chua performed the experiments and analysed the data. Both authors contributed to the writing and reviewing of the manuscript. Both authors approved the final manuscript.

## CONFLICT OF INTEREST

The authors declare no conflict of interest.

## Data Availability

Data available on request from the authors.
